# *Clinacanthus nutans* genetic diversity and its association with anti-apoptotic, antioxidant, and anti-bacterial activities

**DOI:** 10.1038/s41598-023-46105-z

**Published:** 2023-11-10

**Authors:** Salinee Chiangchin, Saruda Thongyim, Hataichanok Pandith, Thida Kaewkod, Yingmanee Tragoolpua, Angkhana Inta, Santi Watthana, Wittaya Pongamornkul, Siriphorn Jangsutthivorawat, Aussara Panya

**Affiliations:** 1 Department of Biology, Faculty of Science, Chiang Mai University, 239, Hauy Kaew Road, Muang District, Chiang Mai, 50200 Thailand; 2 National Extracts and Innovative Products for Alternative Healthcare Research Group, Chiang Mai University, Chiang Mai, 50200 Thailand; 3 Office of Research Administration, Chiang Mai University, Chiang Mai, 50200 Thailand; 4 School of Biology, Institute of Science, Suranaree University of Technology, Nakhon Ratchasima, Thailand; 5 Queen Sirikit Botanic Garden Organization, Chiang Mai, 50200 Thailand

**Keywords:** Genetics, Molecular biology, Plant sciences

## Abstract

*Clinacanthus nutans* (Burm. f.) Lindau has been extensively utilized in Thai folk medicine. However, there has been no prior exploration of its genetic diversity or its correlation with biological activity and phytochemical profiles. Herein, a total of 10 samples of *C. nutans* were collected from different geographic locations in different environments of Thailand, encompassing Northern, Northeastern, and Central regions. The genetic diversity study using sequence-related amplified polymorphism (SRAP) markers showed that all *C. nutans* samples were closely related, as indicated by UPGMA cluster analysis. When comparing the biological activities of *C. nutans* extracts, our findings demonstrated that those sourced from Northern Thailand exhibited the most potent activity in reducing lipopolysaccharide-inducing cell death, as accessed by cell viability assay. Furthermore, they showed remarkable antioxidant and antibacterial activities against *Staphylococcus epidermidis*, *Staphylococcus aureus*, *Pseudomonas aeruginosa*, and *Escherichia coli*. High-performance liquid chromatography (HPLC) analysis of phytochemical profiles revealed consistent chromatography peak patterns across all *C. nutans* extracts. However, they exhibited varying levels of phenolic contents, as judged by the Folin-Ciocalteu assay, which positively correlated with their observed activities. In conclusion, this study highlights the limited genetic variation within *C. nutans* population in Thailand. Furthermore, it underscores the association between the biological activity and the total phenolic contents which might be mainly impacted by environmental conditions.

## Introduction

Natural products derived from various sources such as plants, animals, and fungi have been used in therapeutic approaches for treating several diseases in folk medicine for more than centuries. The bioactive substances originating from herbs and medicinal plants have been continuously receiving great attention and made substantial contributions to modern medicine^1,2^. These compounds, metabolites, and secondary metabolites potentially serve as starting materials for drug synthesis^2^. To date, bioactive ingredients derived from medicinal plants account for approximately 25% of prescription pharmaceuticals, especially as anti-microbial and anti-cancer agents.^3^.

Southeast Asia is a well-known hotspot for plant diversity richness according to the diverse climate of this region^4^. Local communities have long utilized herbs and medicinal plants for therapeutic purposes since ancient times revealing the potential of traditional medicine and its application in modern pharmaceutical research and development (R&D)^2^. The Acanthaceae family, found mainly in tropical and subtropical regions across Southeast Asia, is among the most diverse plant families, containing over 4,300 species^3^. *Clinacanthus nutans* (*C. nutans*) Lindau belonging to the Acanthaceae family has traditionally been employed for treating conditions such as skin rashes, insect bites, and diabetes mellitus in Indonesia, Malaysia, and Thailand^5^. Notably, *C. nutans* is included in Thailand's national list of essential herbal medicines (NLEM) (2016) and is recommended for treating aphthous ulcers, insect bites and herpes simplex virus, varicella-zoster virus infections. Currently, extensive research has unveiled the pharmacological activities of *C. nutans* extract including antioxidant, anti-inflammatory, anti-diabetic, anti-bacterial (i.e., *Bacillus cereus*, *Escherichia coli*, *Salmonella enterica*)^6^ and anti-virus activities (i.e., herpes simplex virus^7,8^, varicella-zoster virus^9^, dengue virus^10^). However, despite these insights into its biological activity, only a limited number of studies have investigated the genetic diversity of *C. nutans* whereas its correlation to the phytochemical profile and biological activity has never been explored.

Understanding the genetic diversity and its impact on the bioactivity of plant extracts is important for crop development. Genetic markers linked to quantitative traits and biological activity can serve as selection markers for plant breeders to improve the productivity and pharmacological properties of the plant^11^. Therefore, this study aims to investigate the relationship between the genetic diversity of *C. nutans* collected from the different regions and their biological activities. We investigated the genetic diversity of *C. nutans* samples from the different regions with various climate environments in Thailand including Northern, Northeastern, and Central Thailand. Ten samples were investigated and compared for their biological activities including anti-cell death activity against LPS-induced endothelial cell death, antioxidant activity, and antibacterial activity. Additionally, we compared the phytochemical profiles and phenolic contents of *C. nutans* extracts. This comprehensive understanding of the genetic profile and its correlation with biological function would contribute to the promotion, enhancement, and sustainable conservation of *C. nutans* for therapeutic and commercial applications.

## Results

### Genetic diversity of *C. nutans* in Thailand

**Figure 1:**
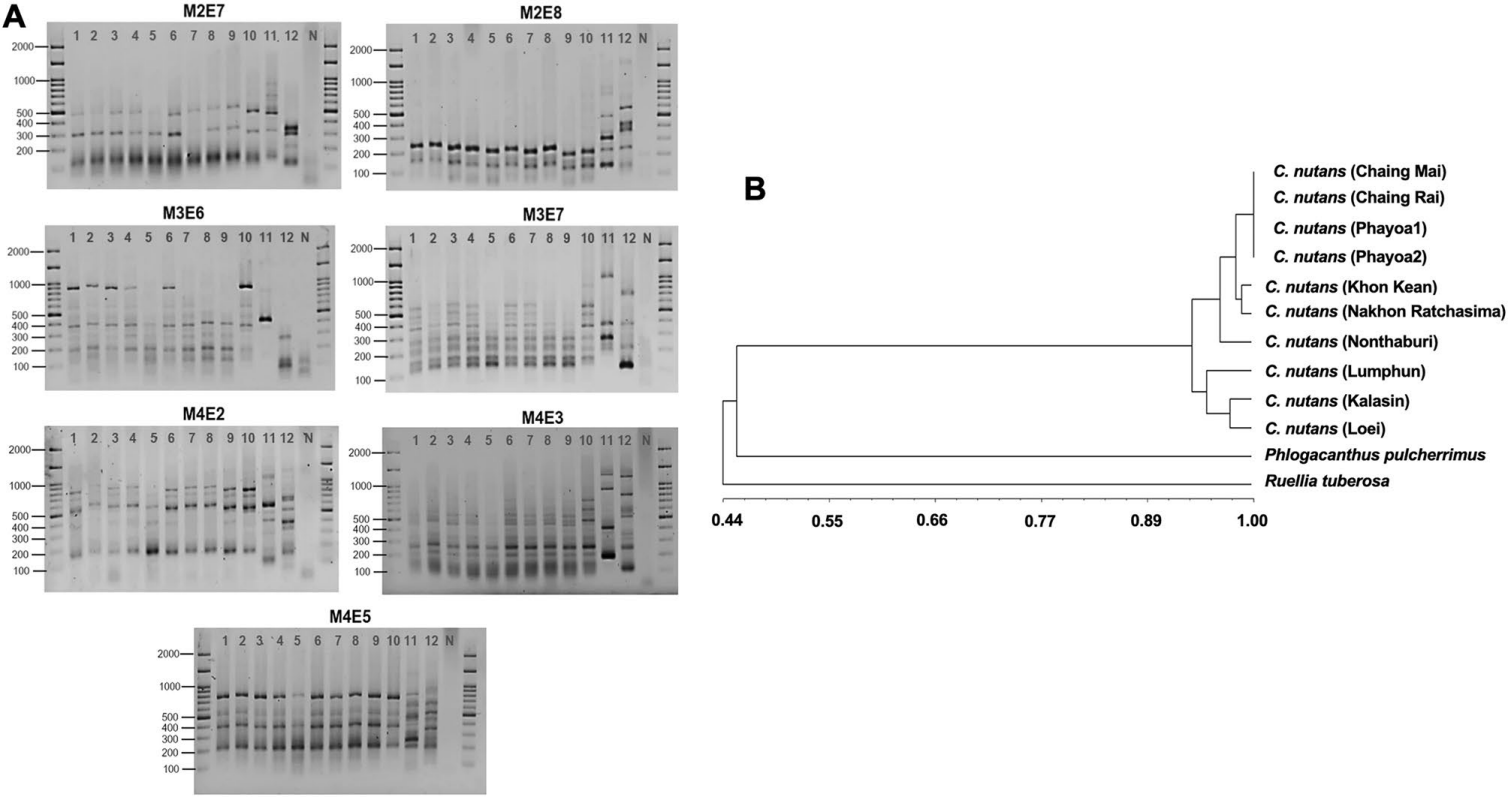
Genetic diversity of *C. nutans.* (A) SRAP technique using 7 pairs of primers (Lane 1–10: *C. nutans* from Chiang Mai, Chiang Rai, Phayoa1, Phayoa2, Lamphun, Khon Kaen, Nakhon Ratchasima, Kalasin, Loie and Nonthaburi; Lane 11: *P. pulcherrimus*; Lane 12: *R. tuberosa*) (**B**) Dendrogram of UPGMA cluster analysis.

Genetic diversity greatly contributed to the bioactivity of the plant extract, particularly in relation to the content of bioactive compounds. We collected the *C. nutans* samples from 10 different regions, including 5 regions in Northern Thailand, 4 regions in the Northeastern part of the country, and 1 region in Central Thailand, each exhibiting variations in either geographic locations or environmental conditions (Table 1). The assessment of genetic diversity was determined using the SRAP technique, coupled with UPGMA cluster analysis. Seven pairs of SRAP markers revealed consistent patterns among *C. nutans* samples from 10 regions. Notably, these patterns were remarkably different from the outgroup, which included *Phlogacanthus pulcherrimus* and *Ruellia tuberosa,* both of which belong to the Acanthaceae family, like *C. nutans* (Fig. 1A, with a full-length gel provided in Supplementary Fig. 1). Furthermore, the UPGMA cluster analysis confirmed the close genetic relationship among *C. nutans* samples, allowing for their classification into 2 distinct divisions, each with a similarity coefficient of 0.95 (Fig. 1B). The first division was composed of Chiang Mai, Chiang Rai, Phayao, Khon Kean, Nakhon Ratchasima, and Nonthaburi provinces, while the other group included Lamphun, Kalasin, and Loei provinces. This technique effectively distinguished the *C. nutans* from the outsource group, yielding a UPGMA coefficient of 0.44.

**Table 1:** List of *C. nutans* collected from 10 different regions of Thailand.

Sample	Province	DMS latitude	DMS longitude	Crude extract (g)	% Yield
A	Chiang Mai	18° 47′ 46.1148″ N	98° 58′ 45.3468″ E	6.63	26.52
B	Chiang Rai	19°54′25.8″ N	99°49′51.44″ E	7.75	31.00
C	Phayao Field 1	19°12′55.57″ N	100°12′8.53″ E	6.00	24.00
D	Phayao Field 2	19°12′55.57″ N	100°12′8.53″ E	4.27	17.08
E	Lumphun	18° 34′ 28.0596″ N	99° 0′ 31.3920″ E	5.05	20.20
F	Khon Kean	16° 26′ 22.6500″ N	102° 49′ 43.4208″ E	7.45	29.80
G	Nakhon Ratchasima	14° 58′ 47.6400″ N	102° 5′ 51.9756″ E	4.24	16.96
H	Kalasin	16°26′18.63″ N	103°30′21.96″ E	6.64	26.56
I	Loei	17°29′9.68″ N	101°43′20.28″ E	6.27	25.08
J	Nonthaburi	13° 51′ 32.7888″ N	100° 31′ 17.9472″ E	3.91	15.64

### Anti-cell death activity of *C. nutans* extract in LPS-induced endothelial cells

**Figure 2:**
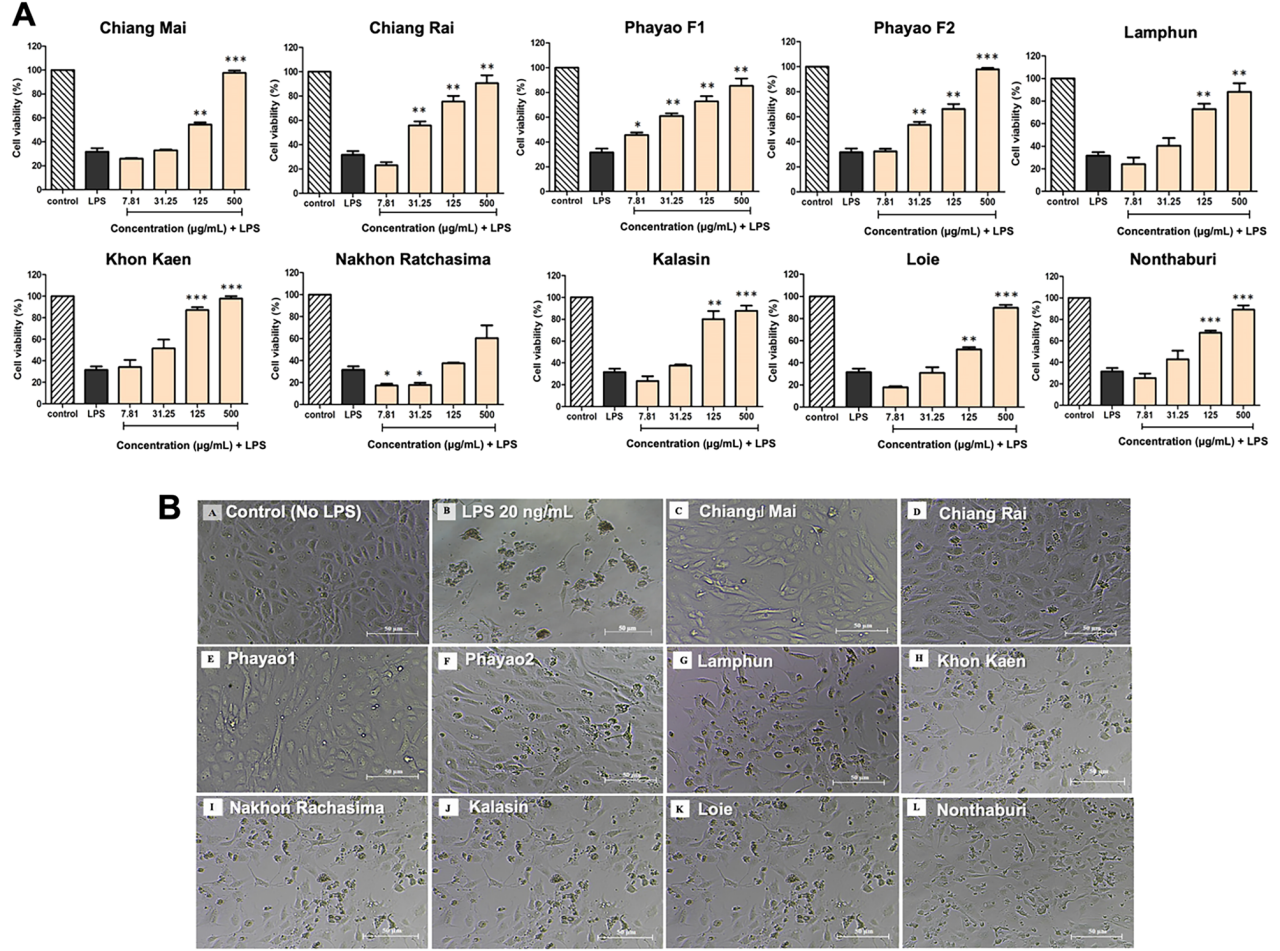
Anti-cell death activity of *C. nutans* extracts. The ethanol extracts of *C. nutans* were determined for their ability to prevent the cell death caused by LPS. CPAE bovine endothelial cells were treated with 20 ng/mL of LPS in absence or presence of *C. nutans* extracts (7.81–500 μg/mL). (**A**) The percentage of cell viability was monitored after 24 h of treatment relative to non-treatment control (set as 100%) (*p < 0.05; **p < 0.01, ***p < 0.001). (**B**) The cell morphology was observed after 24 h of treatment.

To compare the biological activity of *C. nutans* samples, the ethanol extracts were tested for their protective activity against LPS-induced cell death in bovine endothelial cells. Our previous study reported a novel biological function of *C. nutans* in lowering the effect of LPS in the bovine mastitis model^12^. Sublethal doses, as determined in cytotoxicity tests, were used in the experiments with bovine endothelial CPAE cells (Supplementary Fig. 2). In accompanied to our earlier findings, *C. nutans* extracts from 10 different regions exhibited significant potential to neutralize the effects of LPS (Fig. 2). LPS treatment at the concentration of 20 ng/mL dramatically caused cell death, reducing cell viability to less than 25% after 24 h of exposure (Fig. 2A). However, treatment with *C. nutans* extract was able to inhibit the LPS-induced cell death, increasing cell viability to more than 80% at the highest tested concentration (500 μg/mL), except for the extract from Nakhon Ratchasima (Fig. 2A,B). When comparing the effectiveness among *C. nutans* extracts, those from the Northern region of Thailand, including Chiang Mai and Phayao, demonstrated the greatest potential to inhibit more than 50% cell viability at a concentration as low as 31.25 μg/mL.

### Antioxidant activity of *C. nutans* extract

The antioxidant activity of *C. nutans* extract is well-recognized. To compare the antioxidant properties of these extracts, the DPPH assay was performed and calculated for the half-maximal inhibitory concentration (IC50) using non-linear regression analysis. The result confirmed the presence of free radical scavenging capacity in all extracts, although the activity levels varied across different sources (Table 2). The IC50 values ranged from 2.559 ± 0.378 (Chiang Mai) to 9.219 ± 0.210 (Kalasin). Notably, the extract from Chiang Mai exhibited the highest activity; with no statistically significant differences observed when compared to extracts from Chiang Rai (IC50 value of 2.799 ± 0.454), Loei (IC50 value of 3.019 ± 0.092), and Nonthaburi (IC50 value of 3.059 ± 0.270).

**Table 2:** The antioxidant DPPH activity of *C. nutans* extracts.

Sample	Extract sources	DPPH IC_50_ (mg/ml)
A	Chiang Mai	2.559 ± 0.378
B	Chiang Rai	2.799 ± 0.454
C	Phayao Field 1	4.445 ± 0.198
D	Phayao Field 2	4.385 ± 0.555
E	Lumphun	5.128 ± 0.423
F	Khon Kean	4.821 ± 0.012
G	Nakhon Ratchasima	7.280 ± 0.233
H	Kalasin	9.219 ± 0.210
I	Loei	3.019 ± 0.092
J	Nonthaburi	3.059 ± 0.270

### Antibacterial activity of *C. nutans* extract

Previous research has already reported the anti-bacterial activity of *C. nutans* in inhibiting the growth of certain bacteria and yeast including *Bacillus cereus*, *Escherichia coli*, *Salmonella enterica* serovar Typhimurium, and *Candida albicans*^13^. In our study, we aimed to compare the activities of *C. nutans* extracts in inhibiting the growth of *Staphylococcus epidermidis*, *Staphylococcus aureus*, *Pseudomonas aeruginosa*, and *Escherichia coli,* which are common opportunistic pathogens in humans. Among 10 extracts, those from Chiang Mai province conferred the most potent activity against *S. epidermidis*, *S. aureus*, *P. aeruginosa*, and *E. coli* with MIC values of 31.25, 62.5, 62.5, and 31.25 mg/mL, respectively. Furthermore, MBC values for these extracts were determined to be 250, 500, 250, and 500 mg/mL, respectively (Table 3).

**Table 3:** The antibacterial activity of *C. nutans* extracts against *S. epidermidis*, *S. aureus*, *P. aeruginosa*, and *E. coli* judged by MIC and MBC evaluation. Data are given as mg/mL for plant extracts.

Sample	Extract sources	*S. epidermidis*		*S. aureus*		*P. aeruginosa*		*E. coli*	
		MIC	MBC	MIC	MBC	MIC	MBC	MIC	MBC
A	Chiang Mai	31.25	250	62.5	500	62.5	250	31.25	500
B	Chiang Rai	62.5	250	62.5	500	62.5	500	62.5	500
C	Phayao Field 1	31.25	500	31.25	500	62.5	500	62.5	500
D	Phayao Field 2	62.5	500	31.25	500	125	500	125	250
E	Lumphun	125	500	125	500	125	500	250	500
F	Khon Kean	125	500	125	500	125	250	250	500
G	Nakhon Ratchasima	62.5	250	62.5	250	62.5	250	125	250
H	Kalasin	62.5	500	125	500	125	500	31.25	500
I	Loei	125	500	125	500	125	500	62.5	500
J	Nonthaburi	125	500	125	500	125	500	62.5	500
	Gentamicin	0.0156	0.0156	0.0156	0.0156	0.0156	0.0156	0.0156	0.0156

### Phytochemical profiles of *C. nutans*

**Figure 3:**
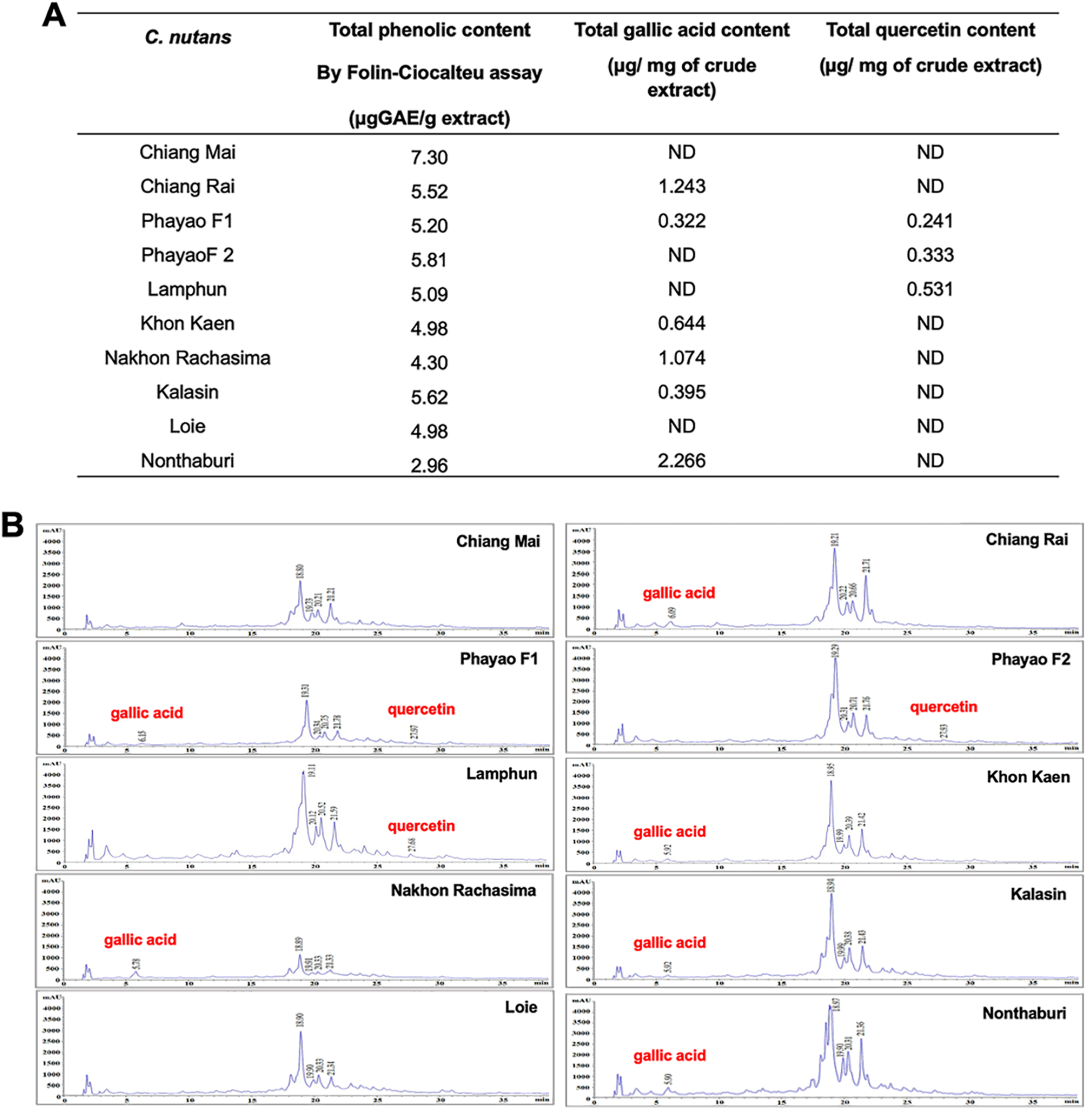
Phytochemical analysis. (**A**) The ethanol extracts of *C. nutans* were determined for their phenolic contents using Folin-Ciocalteu assay. (**B**) Phytochemical profile of the extracts was evaluated using HPLC with a mobile phase consisting of H_2_O containing 0.5% glacial acetic acid (solvent A) and methanol (solvent B). The pure gallic acid and quercetin were used as references to identify and calculate the gallic acid and quercetin contents in the extracts.

While the genetic profiles revealed a close genetic relationship among 10 *C. nutans* samples, the biological activities of these samples varied across different function assays. To elucidate the connection between biological compounds and biological activities, we conducted an investigation of the phytochemical profiles of *C. nutans*. The extracts were determined for their total phenolic contents using the Folin-Ciocalteu assay. The result showed the extracts from the Northern region had, on average, higher total phenolic content compared to those from Northeastern, and Central regions of Thailand (Fig. 3A). The highest total phenolic content was found in the extract from Chiang Mai province (7.30 μg GAE/g extract), whereas the lowest content was observed in the extract from Nonthaburi (2.96 μg GAE/g extract). Furthermore, the profile was characterized using HPLC (Fig. 3B). The HPLC chromatogram exhibited a consistent pattern, but differences were observed in the area under the curves, particularly in the case of four major peaks (indicated by retention times). When we determined the gallic acid and quercetin which were the common active ingredients in plants, interestingly, we observed their presence in relatively low amounts under our tested conditions and mobile phase (Fig. 3A,B) suggesting that the gallic acid and quercetin may not be the major phenolic compounds in *C. nutans* extracts.

## Discussion

*C. nutans* is widely distributed in tropical Asia, where it has been originally found^5^. Although *C. nutans* has diverse biological activity (i.e., antioxidant, anti-inflammation, anti-diabetes, anti-bacterial, anti-viral activities) as well as its long history of safe traditional use, unfortunately, there is a limited availability of commercial *C. nutans* products in the market. These products are primarily utilized for treating the clinical symptoms of herpes simplex virus and varicella-zoster virus infection^14^. Currently, although the biological activities of *C. nutans* have been extensively studied, none of these studies have explored the relationship between its genetic background, phytochemical profile, and biological activity^5^. In this present study, we aim to provide insight into the genetic background, phytochemical profile, and biological activity of *C. nutans* obtained from 10 different regions in Thailand.

We conducted an investigation into the genetic polymorphism of *C. nutans* using DNA fingerprinting, a highly effective and straightforward technique for studying genetic variations^15^. The genotype of a living organism naturally influences its phenotypic traits. However, even slight genetic differentiation and/or variation can have significant implications for quantitative traits within populations. Understanding the naturally occurring genetic variation and its impact on the bioactive compound compositions and/or biological activities is vital for the breeding programs aimed at enhancing productivity, quality, and the sustainable conservation of *C. nutans*. In our study, *C. nutans* samples collected from 10 regions in Thailand were characterized using SRAP technique which was used to amplify coding regions of DNA. SRAP technique is the potential tool applied in various aspects of plant biology, including agronomic and horticultural purposes, analysis of linkage map construction, and identification of quantitative trait loci for advanced hybrids^11,16^. In comparison to the other dominant markers like inter-simple sequence repeat (ISSR), random amplified polymorphic DNA (RAPD), and amplified fragment length polymorphism (AFLP), SRAP markers have the advantage of efficiently elucidating genetic variation at different taxonomic levels with lower cost, reduced technique complexity, and high reproducibility^11,17^. Our results demonstrated seven pairs of SRAP markers effectively distinguished *C. nutans* from outgroup species, including *Phlogacanthus pulcherrimus* and *Ruellia tuberosa* which belong to the same Acanthaceae family. This indicates the potential applicability of SRAP in identifying of *C. nutans* from the other species, especially in case where misidentification occurs due to closely related morphological and test characteristics or when identification becomes challenging after the drying process. However, the genetic fingerprint of the 10 *C. nutans* samples revealed homogeneity with a similarity coefficient of 0.95 (Fig. 1B). This aligns with previous findings, as *C. nutans* has been reported to exhibit high genetic homogeneity^18^. In agreement with our results, Fong and colleagues reported low genetic variation in *C. nutans* among 12 samples collected from Malaysia, Vietnam, and Thailand, using techniques such as restriction fragment length polymorphism (RFLP), RAPD, and microsatellite marker^18^. The primary reason for the low variation of *C. nutans* is its common propagation method among local communities, involving stem cutting (vegetative propagation), which results in genetically identical plant replicas.

Based on our findings using the SRAP technique, low genetic variation was observed among *C. nutans* samples from different locations. This suggests that genetic variation is unlikely to have a substantial impact on the biological activity of *C. nutans*. However, it is important to note that we cannot conclude that genetic diversity has no effect on biological activity, as there are limitations associated with the technique we used, including its sensitivity and the sample size. The SRAP marker is a dominant marker; thus, SRAP amplicons cannot yield heterozygosity descriptors that could directly influence the patterns of the diversity^11^. Recent research has utilized the random amplified microsatellite polymorphism (RAMP) technique, which is a co-dominant molecular marker, to evaluate the genetic diversity of 80 *C. nutans* accessions in Malaysia at the population levels^15^. Therefore, the combined use of SRAP with other markers such as RAPD, AFLP, RAMP, etc. could offer benefits in elucidating greater levels of variation, especially within a group of highly related populations.

The extensive biological activity spectrum of *C. nutans* has been well-documented, indicating the potential in diverse areas, encompassing both infectious (anti-bacterial and anti-virus activity) and noninfectious diseases (anti-inflammation, antioxidant, and anti-diabetes). In our study, we determined the anti-cell death, antioxidant, and anti-bacterial activities and compared the efficacy of *C. nutans* extracts derived from 10 different regions. We demonstrated the anti-bacterial activity of *C. nutans* against common pathogenic bacteria, including *S. epidermidis, S. aureus, P. aeruginosa*, and *E. coli* (Table 3), thereby confirming the efficacy of the extract as an anti-bacterial agent. Recent research has highlighted the protective effect of *C. nutans* extract in a hepatitis B virus (HBV) mouse model^19^. Administration of *C. nutans* in HBV mouse model not only lowered the expression level of HBV antigen but caused the alteration of gut microbiota evidenced by a significant decrease in the proportion of *Lactobacillaceae* and *Lactobacillus*, accompanied by a substantial increase in the relative abundance of *Bacteroidales_S24-7* group, *Rikenellaceae*, and *Alistipes*. It is important to note that while the impact of *C. nutans* extract on the alteration of these bacterial strains was observed, there is currently no direct evidence demonstrating the direct effect of the extract on the growth of these bacteria. It is plausible that the observed result may be attributed to cumulative effects within the gut microbiota community. However, these findings along with metabolomic results (demonstrating the upregulation of hippuric acid, L-histidine, trehalose, D-threitol, and stachyose, and downregulation of uridine 5′-diphosphate, cholic acid, trimethylamine N-oxide, CDP-ethanolamine, and phosphorylcholine) underscore the effectiveness of *C. nutans* in reducing HBV clinical symptoms and conferring protective effects in HBV model mice. This suggests the potential application of *C. nutans* in controlling bacterial growth, which may extend to various diseases, not only bacterial infection but also those related to gut microbiota disorders such as anti-HBV infection.

In addition to its anti-bacterial activity, *C. nutans* treatment exhibited a protective effect against LPS-induced cell death in bovine endothelial cells (Fig. 2) and demonstrated antioxidant activity, as accessed by DPPH assay (Table 2). Oxidative stress, characterized by the excessive production of reactive oxygen or nitrogen species (ROS/RNS), has been extensively documented to play a crucial role in cell death. Elevated ROS/RNS levels in cells lead to lipid membrane disruption, increased fluidity and permeability, protein dysfunction, and aggregation, as well as DNA damage, ultimately resulting in cell damage and apoptosis. This oxidative stress is implicated in the pathogenesis of various diseases, including metabolic diseases, cancer, and other chronic diseases^20^. Considering the common mechanism, it is reasonable to infer that the anti-oxidant activity plays a role in the anti-cell death properties of *C. nutans* extract. On the other hands, the anti-bacterial and anti-oxidant activities has been reported to correlate with the polyphenolic levels of the extract^21^. In our experiments, when comparing the biological effectiveness of 10 *C. nutans* extracts, the extract from the Northern part of Thailand demonstrated the highest biological potency compared to the extracts from other regions. Specifically, extracts from Chiang Rai (DPPH IC50 = 2.799 ± 0.454) exhibited the greatest efficacy in protecting the bovine endothelial cells from LPS-induced cell death (Fig. 2). Additionally, extracts from Chiang Mai (DPPH IC50 = 2.559 ± 0.378) had the highest levels of anti-bacterial activities, as determined by MIC/MBC technique (Table 3).

The evaluation of phytochemical compounds using the Folin-Ciocalteu assay to determine the phenolic content revealed that extracts from the Northern regions contained higher phenolic content than those from other regions. Notably, the extract from Chiang Mai exhibited the highest content, measuring 7.30 μg GAE/g extract (Fig. 3A) suggesting that the biological activity of *C. nutans* extract is associated with the bioactive contents present in each extract. Furthermore, we investigated the phytochemical profiles of these extracts, comparing them across 10 regions using HPLC. Our results indicated that all extracts shared a common profile of chromatogram but varied in terms of quantity based on the peak area. We used gallic acid and quercetin, which have been reported as common phenolic compounds of *C. nutans,* as the references^22,23^. However, our result revealed that gallic acid and quercetin were not the major compounds in *C. nutans* extracts and were not detectable in some extracts, at least under the conditions we tested (Fig. 3).

Total phenolic and flavonoid contents were positively associated with the biological activity of *C. nutans* extract, especially the terms of antioxidant activity^22^. The amount of gallic acid and quercetin can vary depending on the metabolite content itself, as well as the extract method and extraction solvent used. For instance, extractions of *C. nutans* with hot aqueous, aqueous, and aqueous methanol contained sub-detectable levels of gallic acid although these extracts performed the highest DPPH radical scavenging activity when compared to other extract solvents such as hexane, ethyl acetate, absolute methanol^24^. On the other hand, other phenolic compounds were confirmed in these extracts, including protocatechuic acid, chlorogenic acid, ferulic acid, and caffeic acid^24^, which may contribute to the antioxidant activity. In our study, gallic acid and quercetin were found as minor compounds in our extracts, they may not be suitable as biomarkers for pharmacological selection. However, it is important to note that the contribution of these compounds to the activity of *C. nutans* cannot be entirely ruled out, as they may synergistically with the other phenolic and flavonoid compounds in the extract. Alternatively, the basic measuring of total phenolic/flavonoid content serves as a reasonable indicator, at least for assessing antioxidant properties.

Ghasemzadeh and colleagues previously reported on the relationship between the pharmacological activity of *C. nutans* and the age of the plant^22^. Their study compared plants with ages ranging from 1 month old to 1 year old and observed that plant age influenced the variation in photochemical synthesis. Specifically, the age of plant appeared to affect the phytochemical contents of buds, with 6-month-old *C. nutans* buds showing the highest amount of total phenolic content (18.21 ± 1.12 mg/g dry weight) and total flavonoid content (6.32 ± 0.74 mg/g dry weight). However, the age of the plant had a relatively lesser effect on the leaves, whether they were 1 month, 6 months, or 1 year old, with the total phenolic content ranging from 7.29 ± 0.80 to 11.32 ± 1.2 mg/g dry weight, and total flavonoid content ranging from 3.79 ± 0.29 to 4.66 ± 0.43 mg/g dry weight^22^. Notably, both buds and leaves exhibited a similar trend, with phytochemical content decreasing after 6 months of age. In our study, we did not compare the age of samples, we thus cannot conclude that the variations observed in our study were due to differences in plant age. Further investigations, especially considering factors such as cultivation conditions (e.g., growth period, minerals, stress), and their associations with phytochemical content and biological activity, are necessary to establish the best practices for *C. nutans* cultivation.

## Conclusion

*C. nutans* collected from different regions of Thailand exhibited low genetic variation. The observed differences in their biological activities, which included anti-apoptotic, antioxidant, and anti-bacterial activities, were mainly associated with the level of total phenolic and flavonoid contents within the extracts which were possibly influenced by environmental factors.

## Methods

### Collection of plant materials and extraction

*C. nutans* leaves were collected from different regions of Thailand during 2019–2020. The collection of plant material complied with relevant institutional, national, and international guidelines and legislation. The fresh leaves of cultured plants were collected from Mae Rim District (Chiang Mai Province), Chiang Khong District (Chiang Rai Province), Chiang Kham District (Phayao Province; Phayao Field 1), Li District (Lamphun Province), Sikhio District (Nakhon Ratchasima Province). The others were bought at the local markets from Muang District (Phayao Province; Phayao Field 2), Muang District (Khon Kaen Province), Na Haeo District (Loei Province), Muang District (Kalasin Province), Bamg Yai District (Nonthaburi Province). The plant specimens were identified by botanist Dr. Wittaya Pongamornkul and deposited at the Queen Sirikit Botanic Garden Herbarium (QSBG herbarium) as following voucher numbers; WP8603 (Chiang Mai), WP8604 (Chiang Rai), WP8605 (Phayao Field 1), WP8606 (Phayao Field 2), WP8607 (Lamphun), WP8608 (Khon Kaen), WP8609 (Nakhon Ratchasima), WP8610 (Kalasin), WP8611 (Loei), and WP8612 (Nonthaburi).

The fresh leaves were oven-died at 50 ºC and subsequently ground into a fine powder using a conventional grinder. The extraction procedures followed the established protocol outlined in our previous studies^12,25^. Briefly, the extraction was performed by using 70% ethanol at the ratio of 1:20, with shaking at 160 rpm/minute (room temperature) for 12 h. The extract was filtrated using Whatman No. 1 filter paper and subjected to evaporation in a rotary evaporator. The weight after evaporation was measured to calculate the % yield of the extracts (Table 1). The extracts were stored at 4 ºC until used.

### Sequence-related amplified polymorphism (SRAP) and UPGMA cluster analysis

SRAP technique was performed as described previously^16^. Briefly, DNA was isolated using the NucleoSpin Plant II (MACCHEREY-NAGEL, Germany. Ten nanograms of DNA were used as the template of PCR using High-Purity i-Taq™ PCR core kit (iNtRON Biotechnology, Inc., Korea) and 7 different pairs of primers including M2/E7, M2/E8, M3/E6, M3/E7, M4/E2, M4/E3, and M4/E5 (Supplementary Table 1). The PCR reaction was conducted with steps of initial melting (95 °C 5 min) followed by 5 rounds of denaturation (94 °C 1 min), annealing (35 °C 1 min), and extension (72 °C 2 min). After then, the reaction was continued by 35 rounds of denaturation (94 °C 30 s), annealing (56 °C 30 s), extension (72 °C 2 min) followed by a final extension at 72 °C for 5 min. The PCR product was analyzed by gel electrophoresis using 1.5% agarose gel. The DNA band was visualized using by ImageQuant LAS 500 Chemiluminescent Imaging System (GE, Boston, MA, USA).

### Cell line and cell culture

Bovine endothelial cell line: CPAE, (CCL209TM, ATCC, VA, USA) was cultured in the minimal essential medium (MEM) with 20% (v/v) fetal bovine serum (FBS) supplementation. The cells were incubated at 37 ºC under a 5% CO_2_ humidified atmosphere and subcultured when the cell growth reached 80% confluence.

### LPS treatment and cell viability assay

The effect of LPS on CPAE cell death and the anti-cell death activity of *C. nutans* extracts were determined by using a cell viability assay as previously described^12^. The term “anti-cell death activity” refers to the ability of the extract to prevent cell death triggered by LPS stimulation. In our study, we employed endothelial cells (CPAE), which are highly sensitive to LPS activation and play a crucial role in inflammatory response, to illustrate the capacity of extracts to inhibit LPS-induced cell death. We compared the cell viability between treatment conditions with or without extracts as a measure of their anti-cell death activity. Briefly, CPAE was plated a day before the experiment in a 96-well plate (7,000 cells/well). The LPS derived from *Escherichia coli* (catalog no. L4391, *E. coli* 0111: B4, Sigma-Aldrich, St. Louis, MO, USA) was treated to the CPAE at the concentration of 20 ng/mL in the presence or absence of *C. nutans* extracts at the various concentrations ranging from 7.81 to 500 μg/mL. After 24 h of treatment, the cells were harvested and measured for the cell viability by using PrestoBLUE reagent (Thermo Fisher Scientific, MA, USA) to monitor the reducing capacity of living cells relative to non-treatment control. The changes in absorbance were determined at 570 nm and 600 nm using a microplate reader (EZ Read 2000, Biochrom, Cambridge, UK) and used to calculate the percentage of cell viability (% cell viability) relative to that of non-treatment control which set as 100% following this equation.

$$\% \;{\text{Cell}}\;{\text{viability}} = \left[ {\left( {{\text{OD57}}0 - {\text{OD6}}00} \right){\text{ treated}}\;{\text{cells}}/\left( {{\text{OD57}}0 - {\text{OD6}}00} \right)\;{\text{non}}{-}{\text{treated cells}}} \right] \times {1}00.$$

### DPPH radical scavenging assay

The 2,2-diphenyl-1-picrylhydrazyl (DPPH) radical scavenging assay was performed by the method reported by Prieto et al.^26^ to determine the antioxidative activity of *C. nutans* extracts. The extracts were prepared in methanol at various concentrations and mixed with a 0.1 mM DPPH reagent (Sigma-Aldrich, Germany). The plate was incubated for 20 min in a dark place at room temperature and measured for the absorbance at the wavelength of 517 nm and compared to the standard curve obtained from gallic acid (Sigma-Aldrich, Darmstadt, Germany). The antioxidative activity of *C. nutans* extracts was represented as mg of gallic acid equivalents per gram extract (mg GAE/g extracts).

### Minimum inhibitory (MIC) and minimum bactericidal concentration (MBC) evaluations

Minimum Inhibitory (MIC) and minimum Bactericidal Concentration (MBC) evaluations were conducted as previously described^27^. The MIC values were determined by using the broth dilution method. Standard laboratory strains of bacteria including *Staphylococcus epidimidis* (ATCC 14990), *Staphylococcus aureus* (ATCC 25923), *Pseudomonas aeruginosa* (ATCC 27853) and *Escherichia coli* (ATCC 25922) were kindly obtained from the Microbiology Section, Department of Medical Technology, Faculty of Associated Medical Science, Chiang Mai University, Chiang Mai, Thailand. The bacterial suspension was prepared in McFarland No. 0.5. The *C. nutans* extract (7.8–500 mg/mL) was then added to the bacterial culture. The turbidity reflecting bacterial cell growth was measured after 24 h of incubation at 37 °C. The MBC values was determined by streaking the plate from the tubes in which the bacterial was not visible. The plate was incubated for 24 h at 37 °C to evaluate the MBC endpoint which defined as the lowest concentration that *C. nutans* inhibited up to 99.9% of bacterial growth.

### Folin-Ciocalteu assay

The Folin-Ciocalteu assay was conducted as described by Wolfe et al.^28^ to determine the total phenolic contents in *C. nutans* extracts. Briefly, the extracts were mixed with 50% (w/v) Folin-Ciocalteu reagent (Merck, USA) and incubated for 5 min in a dark place before adding 5% (w/v) sodium carbonate. The reaction was incubated for 1 h and measured for the absorbance at OD725. The gallic acid (Sigma-Aldrich, Darmstadt, Germany) was used as the reference in which the total phenolic contents were calculated and represented as mg of gallic acid equivalents per gram extract (mg GAE/g extracts).

### High-performance liquid chromatography (HPLC)

The phytochemical profiles of *C. nutans* extracts were determined by using gradient HPLC systems with the ZORBAX Eclipse XDB-C18 column (Agilent Technologies, USA) as the protocol described previously^29^. HPLC used a mobile phase consisting of water with 0.5% glacial acetic acid (solvent A) and methanol (solvent B). The gradient steps were conducted at ambient temperature using the following steps: 100% A, 0–20 min; 50% A, 20–30 min; 40% A, 432 30–35 min; 30% A, 35–40 min; 20% A, 40 min; post-time, 5 min before next injection. The flow rate was 1.0 mL/minute, and the injection volume was 20 μL. A UV photodiode array detector (270 nm) was used to monitor the wavelength. The gallic acid and quercetins (Sigma-Aldrich, Darmstadt, Germany) were used as the standard compound to calculate the amount of gallic acid and quercetins in *C. nutans* extracts.

### Statistical analysis

The data from at least three independent experiments were used to analyze the statistical differences using the Student’s t-test (GraphPad Prism version 5.0).

### Supplementary Information

Supplementary Information.

## Data Availability

The data supporting this study's findings are available from the corresponding author upon reasonable request.

## Abbreviations

SRAP: Sequence-related amplified polymorphism
LPS: Lipopolysaccharide
MIC: Minimum inhibitory concentration
MBC: Minimum bactericidal concentration
DPPH: 2,2-Diphenyl-1-picrylhydrazyl
HPLC: High-performance liquid chromatography
