# Supplementary material for: Clinacanthus nutans genetic diversity and its association with anti-apoptotic, antioxidant, and anti-bacterial activities
Source: Sci Rep. 2023 Nov 10;13:19566. doi: 10.1038/s41598-023-46105-z (PMC10638387; doi:10.1038/s41598-023-46105-z)
Supplement: Supplementary file 1 — Supplementary Information. [file 41598_2023_46105_MOESM1_ESM.pdf]

Supplementary data

Supplementary Table 1 The list of primers used in this study.

Primers	Sequence 5´-> 3´	Length (bp)
**Forward**		
**M-2**	TGAGTCCAAACCGGAAG	17
**M-3**	TGAGTCCAAACCGGAAC	17
**M-4**	TGAGTCCAAACCGGAAT	17
**Reverse**		
**E-2**	GACTGCGTACGAATTAAT	18
**E-3**	GACTGCGTACGAATTGAC	18
**E-5**	GACTGCGTACGAATTCAA	18
**E-6**	GACTGCGTACGAATTCAG	18
**E-7**	GACTGCGTACGAATTCAC	18
**E-8**	GACTGCGTACGAATTCTG	18

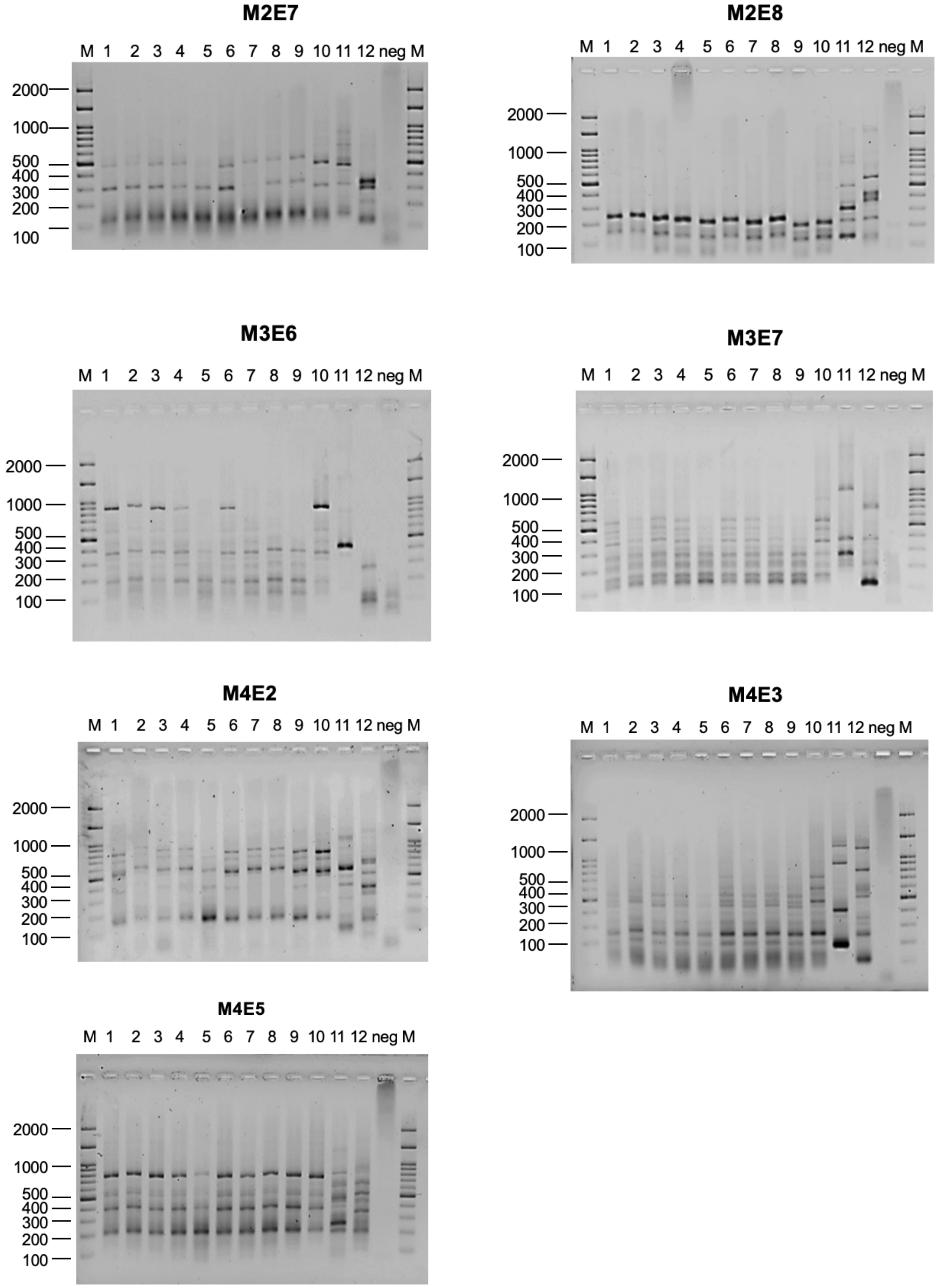

Supplement Figure 1 SRAP technique using 7 pairs of primers; M2E7, M2E8, M3E6, M3E7, M4E2, M4E3, and M4E5. Lane 1-10: *C. nutans* from Chiang Mai, Chiang Rai, Phayoa1, Phayoa2, Lamphun, Khon Kaen, Nakhon Ratchasima, Kalasin, Loie and Nonthaburi; Lane 11: *P. pulcherrimus*; Lane 12: *R.tuberosa*.

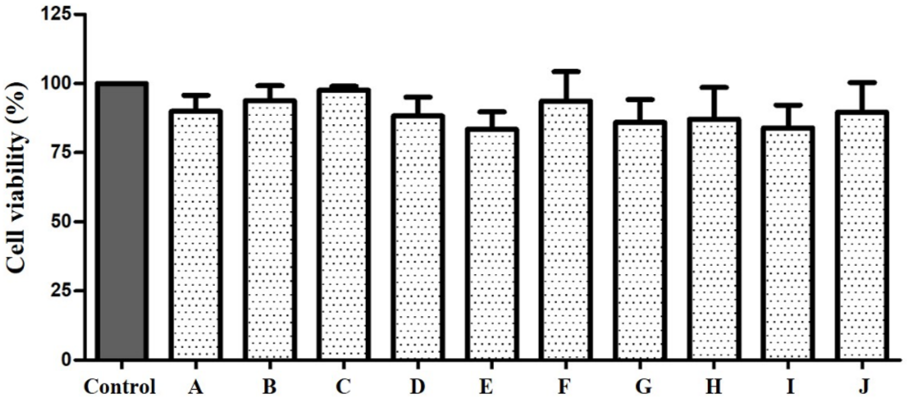

Supplement Figure 2 Cytotoxicity of *C.nutans* extract in CPAE cells. The cell viability of CPAE was determined after treatment of 500 µg/mL of *C.nutans* extract from Chiang Mai (A), Chiang Rai (B), Phayao Filed 1 (C), Phayao Filed 2 (D), Lumphun (E), Khon Kean (F), Nakhon Ratchasima (G), Kalasin (H), Loei (I), Nonthaburi (J) Province.
